# CBCT (Cone-Beam CT)-Based Online Adaptive Radiotherapy in the Post-operative Oesophageal Cancer Patient

**DOI:** 10.7759/cureus.70971

**Published:** 2024-10-07

**Authors:** Amy Ward, Siobhan Graham, Ewan Almond, Dom Withers

**Affiliations:** 1 Oncology, Queen's Hospital, BHRUT (Barking, Havering and Redbridge University Hospitals NHS Trust), Romford, GBR; 2 Radiotherapy, Queen's Hospital, BHRUT (Barking, Havering and Redbridge University Hospitals NHS Trust), Romford, GBR; 3 Radiotherapy Physics, Queen's Hospital, BHRUT (Barking, Havering and Redbridge University Hospitals NHS Trust), Romford, GBR

**Keywords:** adaptive radiation therapy, concomitant chemo-radiation therapy, oesophageal cancer, organ at risk, post operative radiotherapy

## Abstract

We present a case of a patient having cone-beam CT (CBCT)-based online adaptive radiotherapy (oART) on Ethos Therapy after oesophagectomy and gastric pull-up. This case report aims to demonstrate that daily oART is a viable treatment option for post-oesophagectomy patients. The patient's radiotherapy plan was generated on the Ethos system using an eight-field intensity-modulated radiation therapy (IMRT) plan imported from the Eclipse planning system. The patient was prescribed 50.4Gy/28# over 5.5 weeks with concurrent carboplatin and paclitaxel. There were no changes to our department's standard clinical tumour volume (CTV) and planning tumour volume (PTV) margins due to the novel nature of the case. For each fraction of daily oART, the heart, right and left lungs, oesophagus and trachea were Ethos-contoured ‘Influencer’ structures which were reviewed and edited at the console. Minor edits were required to each structure. The adaptive plan was chosen for each fraction due to showing clear dosimetric benefits to both PTV coverage and organ at risk (OAR) doses. Lung doses were significantly reduced with the course mean lung dose reduction of 8.1% and the Lung-CTV V20 reduction of 4.4%. The treatment duration from the start of CBCT acquisition to the end of beam delivery averaged 19 minutes and 12 seconds. The patient was able to tolerate the time on the bed without incident within the scheduled 30-minute appointment slot. Our case demonstrates that significant inter-fraction anatomy changes occur in patients having radiotherapy after oesophagectomy. Without the use of adaption, we have seen that there would be clinically significant undercoverage of target volumes due to sub-optimal dose distribution. We plan to evaluate the imaging available in Ethos dose monitoring to establish if these margins could be reduced in patients treated with online adaptive radiotherapy.

## Introduction

This case report aims to detail experience of treating a patient who required radiotherapy post-operatively following an Ivor Lewis oesophagectomy. Following such surgery, the patient’s oesophagus is removed and the stomach is formed into a gastric tube, which is ‘pulled up’ into the chest to replace it. This gastric pull-up varies in daily filling which is challenging to control with a fasting protocol.

At our institution previously, such patients were treated with image-guided radiation therapy (IGRT). However, marked interfractional anatomical changes led to challenges in image matching and treatment delivery. As such, we decided to deliver kilovolt (kV) cone-beam CT (CBCT)-guided online adaptive radiotherapy (oART) to optimise the treatment plan on each day’s CBCT findings.

The use of online and offline adaptive radiotherapy strategies to treat patients with an intact oesophageal tumour who are medically or oncologically inoperable is not new [1-7]. These strategies all have the aim to optimise the radiotherapy dose distribution in response to changes seen typically, in anatomy, treatment response or patient positioning. Numerous authors have discussed strategies using a range of online and offline adaptive across conventional and magnetic resonance (MR) Linacs. However, to our knowledge, there are no other case reports published to date of using kV CBCT-driven oART for this purpose in the literature. We therefore present our case which demonstrates the benefits of using oART in this novel situation.

## Case presentation

A 52-year-old male presented with epigastric pain and dysphagia to solid food. An endoscopy showed a marked abnormality at the lower oesophagus involving the gastro-oesophageal junction. Biopsies confirmed a poorly differentiated adenocarcinoma, human epidermal growth factor receptor 2 (HER-2) and programmed death ligand 1 (PDL1) negative. Fluorodeoxyglucose-positron emission tomography (FDG-PET) and endoscopic ultrasound were used to complete the AJCC TNM 8th edition staging of T3N2 (peri-oesophageal and peri-gastric nodal involvement) M0.

As routine practice for our UK-based centre, he underwent neoadjuvant chemotherapy with four cycles of FLOT (5FU, Leucovorin, Oxaliplatin, Docetaxel) chemotherapy. This was poorly tolerated, but a good partial response was seen on FDG-PET. The primary tumour reduced from 6 cm to 4 cm in length with a reduction in standardized uptake value (SUV) max from 14.5 to 9.8. He proceeded to Ivor Lewis Oesophagectomy.

He recovered well, but unfortunately post-operative histology demonstrated a positive proximal margin. He was restaged at ypT3ypN3M0. Prior to adjuvant therapy, a further FDG-PET scan was performed which demonstrated a single new right paratracheal lymph node. Due to toxicity of FLOT, it was decided to commence chemoradiation to treat the positive margin and paratracheal lymph node.

Radiotherapy technique

A prescription dose of 50.4Gy in 28 fractions over 5.5 weeks was planned for delivery to the planning tumour volume (PTV), along with concurrent carboplatin and paclitaxel. Three-dimensional free-breathing planning simulation CT scan was performed with intravenous contrast. The patient was positioned supine with one arm up using standard departmental immobilisation with a wing board, knee rest and custom-made vac bag.

FDG-PET scan was used to delineate the paratracheal nodal gross tumour volume (GTV) and site of positive superior margin at the surgical anastomosis. Clinical tumour volumes (CTVs) were grown from both sites, editing from normal tissue as per our departmental treatment protocol. CTV was then expanded by 0.5 cm axially and 1 cm in superior/inferior planes. We did not change CTV to PTV expansion margins from our non-adaptive IGRT protocols due to the novel nature of this case.

An eight-field intensity-modulated radiation therapy (IMRT) beam arrangement was imported from Aria Eclipse v15.6 to Ethos v1.1 as the patient was scanned with one arm down meaning that the default beam arrangements created by Ethos would not be optimal. This imported beam arrangement was then used by Ethos for optimisation.

Table 1 describes the Ethos planning directives, a set of pre-defined clinical goals used to generate a reference plan. The same goals are applied to generate the daily adaptive plan. 

**Table 1:** Ethos clinical planning directives PTV: planning tumour volume, N/A: non-applicable, CTV: clinical tumour volume, PRV: planning organ at risk volume.

Structure	Goal	Variance	Priority
PTV 50.4Gy	V95%≥98%	≥95%	1
	V108%<0.1cc	N/A	1
	Dmax<110%	N/A	2
Both lungs	Dmean<15Gy	N/A	2
Heart	V30Gy≤30%	N/A	2
Lungs CTV	V20≤25%	N/A	2
	V5≤80%	N/A	2
Spinal cord PRV	Dmax<45Gy	N/A	1

The patient plan and datasets were sent to Mobius3D v4.0.1 for pre-treatment quality assurance (QA) where the plan is re-calculated using a collapsed cone algorithm and the resulting dose distribution is compared to that generated by Ethos. A 3%/3 mm gamma criterion with a 10% threshold was used. The plan passed with 98.8% with majority of failure points being within the lung, as expected.

Online adaptive radiotherapy treatment delivery

During each treatment fraction, the patient was set up in the simulated position on the Ethos Linac. A CBCT using Thorax protocol was acquired, reconstructed using HyperSight Acuros algorithm. The imaging was deemed adequate in each fraction to proceed with the workflow.

**Figure 1:**
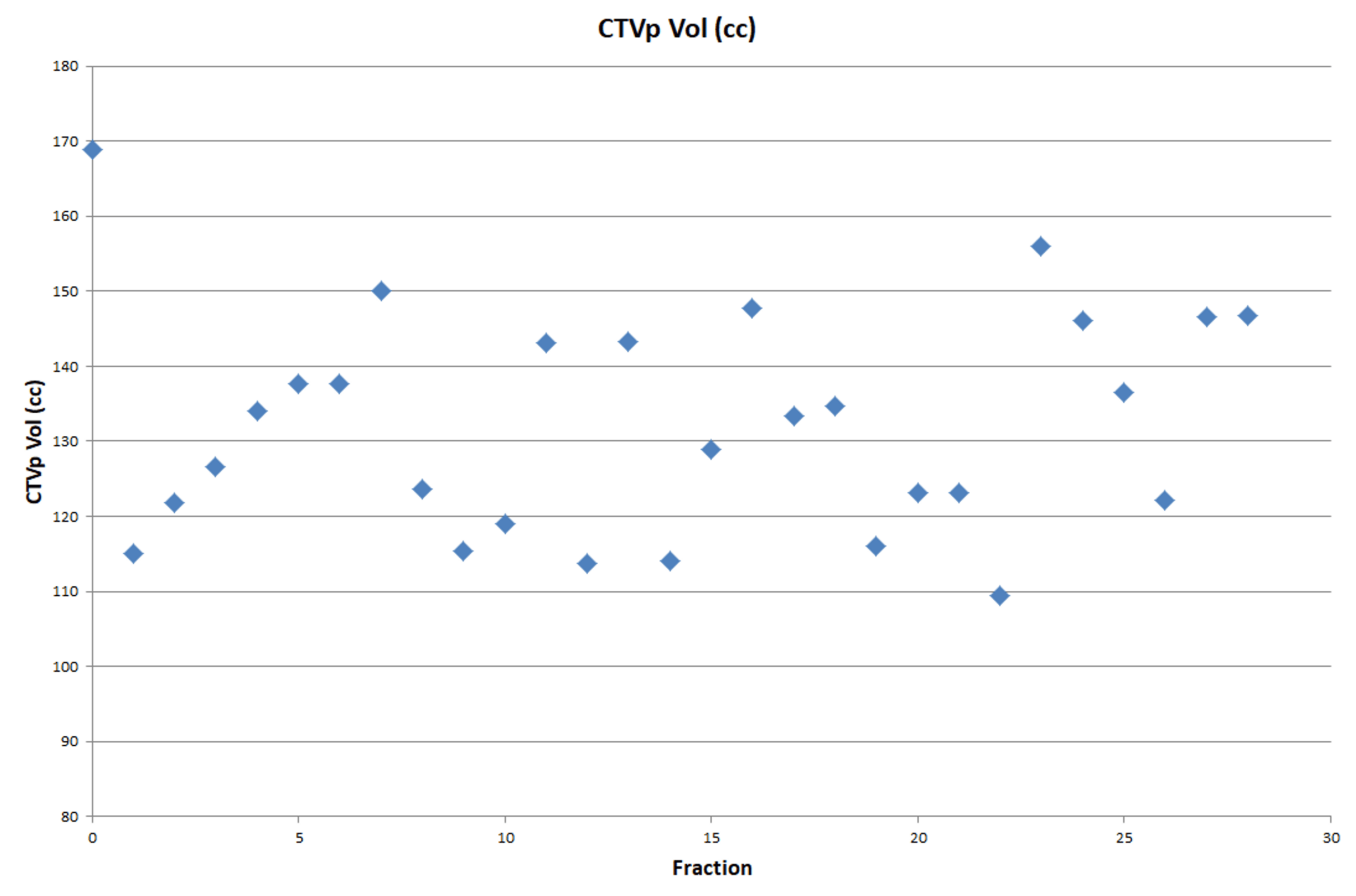
Interfractional change in target volume (gastric pull-up tube) The largest reduction in clinical tumour volume primary (CTVp) volume from the planning scan is 59cc. This was on #22 and correlates with the largest reduction in lung doses when choosing the adaptive plan over the scheduled.

Heart, right and left lungs, oesophagus and trachea were Ethos-contoured ‘Influencer’ structures. These structures are used by the system to propagate the target volumes on the CBCT taken each day. These structures require review and editing at the console. Minor edits were required to each structure. The most changes made were to right lung structure where this abutted the pulled up gastric tube which itself showed notable interfraction changes. Figure 1 demonstrates the volume change across each fraction. CTVs (cropped from lung) and PTVs propagated well, and no corrections were required to target volumes on any of the treatment fractions.

Benefits seen from online adaptive workflow

**Figure 2:**
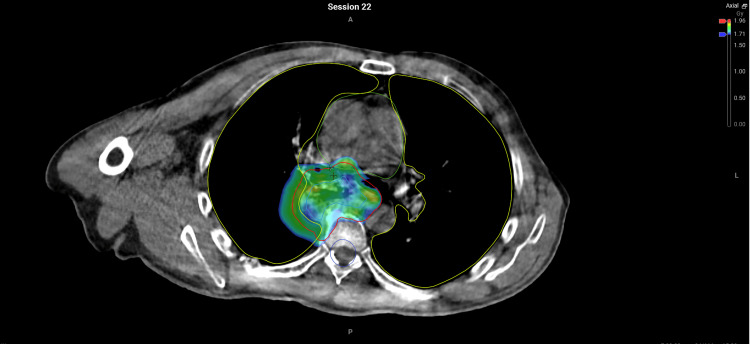
Non-adapted plan demonstrating colourwash of 95% isodose spill into lung #22 Lung V20Gy (V0.71Gy) = 19.8%.

**Figure 3:**
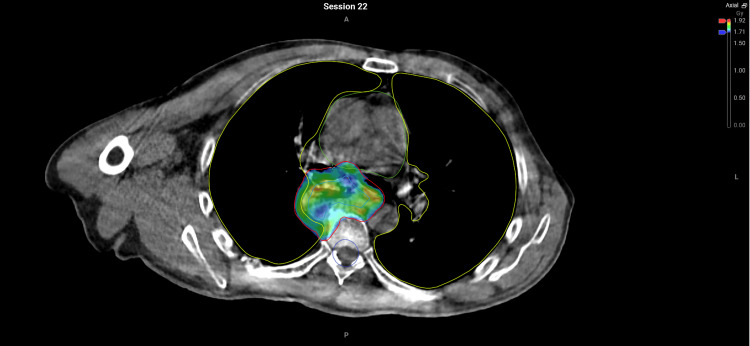
Adapted plan demonstrating colourwash of 95% isodose conforming to the day's anatomy #22 Lung V20Gy (V0.71Gy) = 12.8%.

The adaptive plan showed clear dosimetric benefits and was chosen for each fraction. Lung doses were significantly reduced with the course mean lung dose reduction of 8.1% and the Lung-CTV V20 reducing by 4.4%. The lung dose improvement was most evident on fraction 22 where the CTV primary (CTVp) volume was 59cc less than on the reference CT. Figures 2, 3 when compared demonstrate the reduction in the volume of normal lung tissue being included within the 95% isodose if the adaptive plan was to be chosen.

**Figure 4:**
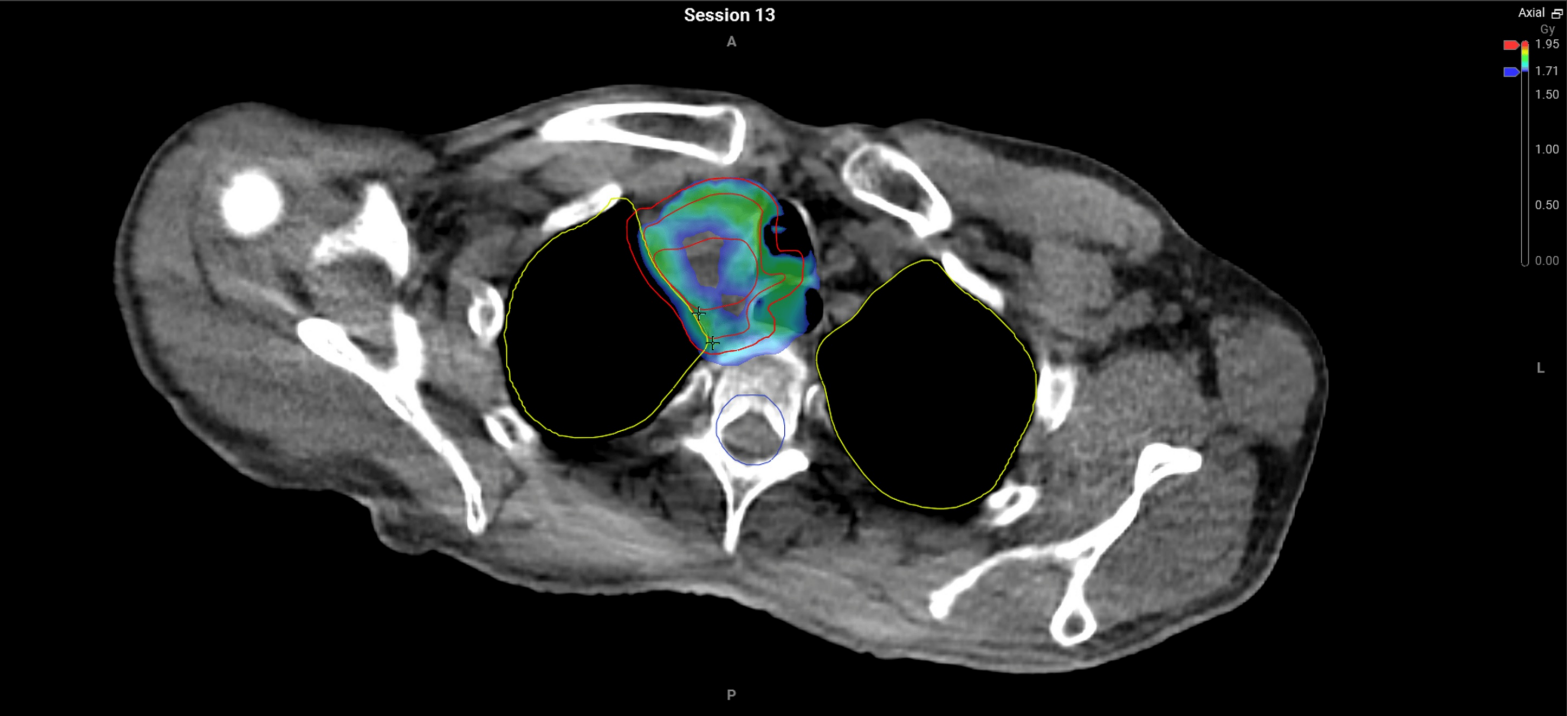
Non-adapted plan demonstrating holes in colourwash of 95% isodose coverage #13 (V95=92.6%).

**Figure 5:**
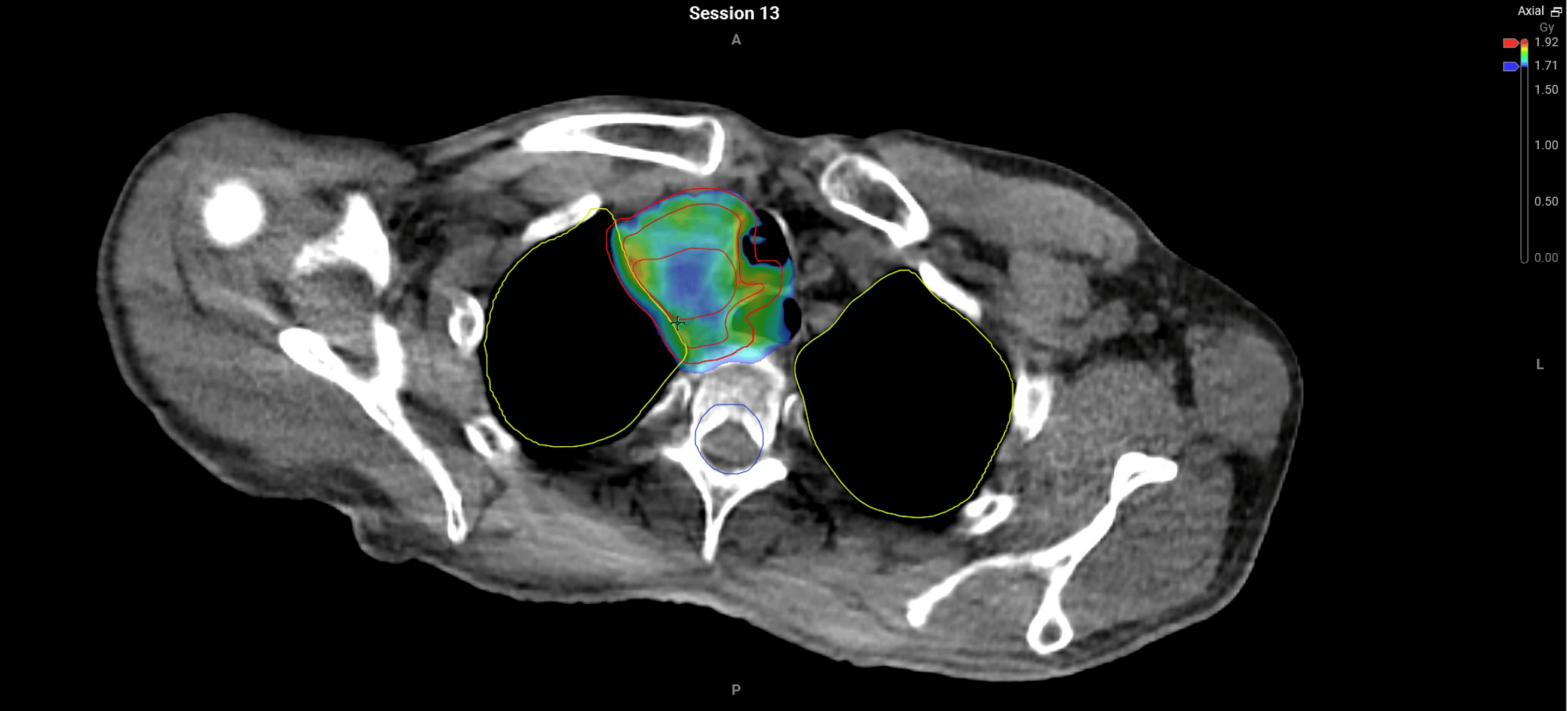
Adapted plan demonstrating superior coverage of 95% isodose when reviewed as colourwash #13 (V95=96.1%).

Typically, the target coverage was improved. In 20 of the 28 fractions, PTV 50.4Gy V95 was greater for the adaptive plan and was always greater than 95%. In four of the 28 fractions, the scheduled plan would have had sub-optimal coverage of the PTV (V95<95%). Figures 4, 5 show this for fraction 13 where the poor coverage was most evident.

The cumulative dose to spinal cord PRV was also reduced in comparison with scheduled plans although did remain within clinically acceptable tolerance for both scheduled and adaptive plans. Given the site of the treatment volume, the heart V40 was not of clinical significance for either plan. However, a minimal reduction in heart V30 was seen on the adaptive plan. Table 2 lists the cumulative doses to the targets and organs at risk (OARs).

**Table 2:** Cumulative doses to targets and OARs PTV: planning tumour volume, CTV: clinical tumour volume, PRV: planning organ at risk volume, OAR: organ at risk.

	Adapted Plan	Non-Adapted Plan
PTV 50.4Gy V95 (%)	97.4	96.8
PTV 50.4Gy Dmax (%)	107.2	108.6
Spinal cord PRV Dmax (Gy)	45.0	47.4
Lung mean dose (Gy)	9.1	9.9
Heart V30 (%)	2.6	3.6
Lung-CTV V5 (%)	46.3	47.8
Lung-CTV V20 (%)	14.8	19.2

The treatment duration from the start of CBCT acquisition to end of beam delivery averaged 19 minutes 12 seconds, ranging from 16 minutes 51 seconds to 20 minutes 50 seconds. The adaptive process took an average of 13 minutes 24 seconds and beam delivery averaged 3 minutes 34 seconds. The patient was able to tolerate the time on the bed without incident within the scheduled 30-minute appointment slot.

## Discussion

Whilst we could not identify any previous cases of post-oesophagectomy radiotherapy delivered with an online adaptive radiotherapy strategy, our case demonstrated similar dosimetric benefits to other authors who deployed offline adaptive strategies in patients with an intact oesophagus [2-4]. There are no studies that compare CBCT-driven oART with MR-guided oART.

Clinical outcome

Treatment was tolerated well. No acute radiotherapy toxicity higher than grade 1 was noted. At last review six months post-radiotherapy, there was grade 1 oesophageal toxicity with some restrictions such as swallowing bread. There was no respiratory toxicity.

FDG-PET scan at three months showed excellent local response with no recurrence at the anastomosis and decreased size and activity of the paratracheal node. Unfortunately, FDG-PET at six months has demonstrated progressive nodal metastasis outside of the treatment volume. This suggests oART has achieved local control with minimal resulting acute or late toxicity but with resulting systemic failure. Ongoing surveillance for the emergence of late toxicity and survival is required.

Previous investigators have used retrospective data to demonstrate mixed clinical outcomes for chemoradiotherapy in the post-operative setting for oesophageal cancer [8-10]. However, no studies are directly comparable to our case of adenocarcinoma with a residual node and R1 resection following neoadjuvant chemotherapy. Raman et al. [8] did identify a survival advantage with adjuvant chemoradiation in R1-resected patients when compared with no adjuvant therapy; however, patients with a history of neoadjuvant treatment were excluded from the review. In unadjusted data of this US-based study, there was a 14% survival rate at five years with the use of adjuvant chemoradiation for R1 resections. Zheng et al. [9] also demonstrated this benefit in a Chinese population, although this cohort was entirely of squamous cell histology and R1 resections were excluded. On the other hand, Fang et al. [10] failed to find a survival benefit in a similar population. None of these retrospective studies were able to comment on radiotherapy-related toxicity.

The overall paucity of data in this area, coupled with limitations of a single patient case in our report, demonstrates that further work is needed to define the benefits of adaptive radiotherapy in this setting. Our case report also lacks long-term survival data. However, when overall outcomes are poor [8], strategies to improve target coverage and reduce radiotherapy toxicity due to favourable OAR dose are crucial.

## Conclusions

Our case demonstrates that significant interfraction anatomy changes occur in patients having radiotherapy post-oesophagectomy. Without the use of adaption, we have seen that there would be clinically significant reduced coverage of target volumes due to sub-optimal dose distribution. We saw a significant reduction in lung (mean and V20) and spinal cord doses by daily adaption to the anatomy. The treatment was comfortably feasible in a 30-minute treatment slot.

Even though this patient has sadly relapsed systemically, good local control has been achieved without problematic acute or late radiotherapy toxicity. This remains important for quality of life even in the context of metastatic disease. As this was a novel case, standard CTV to PTV expansion margins were used. However, we plan to evaluate the imaging available in Ethos dose monitoring to establish if these margins could be reduced in patients treated with online adaptive radiotherapy.
